# NAP1L5 in acute myeloid leukemia: a prognostic biomarker and potential therapeutic target

**DOI:** 10.3389/fonc.2025.1617564

**Published:** 2025-07-25

**Authors:** Meng Wang, Zhibin Xie, Yuanyuan Tan, Yan Zhou, Tingting Zhang, Yuqing Du, Huan Wu, Lili Zhou, Jian Ge

**Affiliations:** ^1^ Department of Hematology, The First Affiliated Hospital of Anhui Medical University, Anhui, Hefei, China; ^2^ Department of Hematology, First Affiliated Hospital of Bengbu Medical University, Anhui, BengBu, China; ^3^ Department of Stomatology, Bengbu Medical University, Bengbu, Anhui, China; ^4^ Department of Psychiatry, Bengbu Medical University, Bengbu, Anhui, China

**Keywords:** acute myeloid leukemia, NAP1L5, biomarker, prognosis, immune infiltration

## Abstract

**Background:**

Nucleosome assembly protein 1-like 5 (NAP1L5), a critical regulator of gene transcription and nucleosome assembly, has been implicated in the progression and poor prognosis of various cancers. However, its specific role and molecular mechanisms in acute myeloid leukemia (AML) remain largely unexplored.

**Methods:**

To identify key genes associated with AML, we analyzed gene expression profiles from AML patients and healthy controls using microarray datasets obtained from the GEO database. Differential expression analysis was performed to identify differentially expressed genes (DEGs), among which NAP1L5 emerged as a critical candidate based on its expression patterns and prognostic relevance, and we validated NAP1L5 expression in clinical AML samples. To elucidate the functional role of NAP1L5, we conducted Gene Set Enrichment Analysis (GSEA) and Gene Ontology (GO) analysis, which revealed its involvement in specific signaling pathways and biological processes. Furthermore, we constructed an interaction network and predictive model for NAP1L5, complemented by an assessment of its role in immune infiltration and drug sensitivity. Finally, we conducted *in vitro* experiments to explore its biological functions and underlying molecular mechanisms.

**Results:**

In AML, elevated expression of NAP1L5 was significantly associated with reduced overall survival, underscoring its prognostic relevance. GSEA revealed that NAP1L5 was prominently enriched in pathways related to apoptosis and DNA replication. GO analysis further indicated that its co-expressed genes were closely linked to autophagy and stress response mechanisms. Interaction network analysis revealed that NAP1L5 engages in complex regulatory interactions with multiple genes, miRNAs, transcription factors (TFs), and RNA-binding proteins (RBPs). Notably, high NAP1L5 expression correlated with increased infiltration of resting CD4+ memory T cells, implicating its potential influence on the tumor immune microenvironment. A predictive model integrating NAP1L5 expression and clinical AML features exhibited robust prognostic utility. Drug sensitivity analysis identified NAP1L5 overexpression as a marker of resistance to Zibotentan, along with associations with 49 additional therapeutic agents. *In vitro* functional assays demonstrated that NAP1L5 overexpression promoted cellular proliferation, migration, and colony formation while concurrently inhibiting apoptosis, highlighting its oncogenic potential in AML pathogenesis.

**Conclusions:**

NAP1L5 emerges as a promising prognostic biomarker and therapeutic target in AML, offering potential for improved patient outcomes and precision treatment strategies.

## 1 Introduction

Acute myeloid leukemia (AML), the most common hematologic malignancy, is characterized by the uncontrolled proliferation of highly heterogeneous hematopoietic stem and progenitor cells, leading to significant variability in prognosis and treatment response (1). Despite recent advancements in clinical diagnosis and targeted therapies, such as inhibitors targeting FLT3, IDH1, IDH2, and BCL2, the five-year survival rate for AML remains below 50%, with many patients experiencing treatment failure and relapse (2, 3). Prognosis in AML is influenced by factors such as patient age, comorbidities, and clinical characteristics, but genetic features of the leukemia play a pivotal role (4). Although traditional “3 + 7” chemotherapy regimens and targeted therapies have improved outcomes for some patients, drug resistance and relapse remain major challenges, often resulting in poor prognosis and severe hematological toxicity (5, 6). Consequently, the development of novel immunotherapeutic or targeted strategies to improve AML prognosis is a critical area of research, and the identification of sensitive and reliable biomarkers is essential to achieving this goal.

Nucleosome assembly protein 1-like 5 (NAP1L5), a member of the NAP1 family, functions as a histone chaperone involved in DNA repair and nucleosome assembly within the nucleus (7). Previous studies have demonstrated its role in transcriptional regulation, DNA replication and repair, chromatin assembly, and demethylation (7, 8). Dysregulated NAP1L5 expression has been implicated in various cancers. For instance, its downregulation in hepatocellular carcinoma (HCC) is associated with poor prognosis (9), while its upregulation in pancreatic ductal adenocarcinoma promotes tumor progression (10). However, the prognostic significance and biological function of NAP1L5 in AML remain poorly understood.

In this study, we identified NAP1L5 as a key gene in AML progression through bioinformatics analysis and explored the molecular mechanisms underlying its aberrant expression in AML prognosis. Furthermore, we validated the functional role of NAP1L5 in AML through *in vitro* experiments. Our findings suggest that NAP1L5 may serve as a novel prognostic biomarker and therapeutic target in AML.

## 2 Methods

### 2.1 Data sources

The AML dataset was obtained from The Cancer Genome Atlas (TCGA)-LAML through the TCGA data portal (https://portal.gdc.cancer.gov/), which included 150 AML samples. Data retrieval and processing were facilitated using the TCGAbiolinks package (11). Corresponding clinical information for the TCGA-LAML samples was collected from the UCSC Xena database (http://genome.ucsc.edu) (12). Additionally, two human AML-related expression profiling datasets—GSE24395 (13) and GSE114868 (14)—were retrieved from the Gene Expression Omnibus (GEO) database using the GEOquery package (15). These datasets comprised 206 AML samples and 25 normal samples. Detailed information on the datasets is provided in **Supplementary Table S1**.

### 2.2 Screening and identification of key differentially expressed genes

In the GSE24395 and GSE114868 datasets, differential expression genes (DEGs) were identified using the limma package. Based on a review of relevant literature and testing various thresholds (16, 17), we applied inclusive thresholds of |logFC| > 0 and P values < 0.05 to identify DEGs. This approach ensured the inclusion of subtle yet potentially significant expression changes, particularly within complex regulatory networks or scenarios where minor alterations might lead to substantial phenotypic effects, thereby maximizing coverage of potentially relevant genes. Volcano plots were generated to visualize the results. Common differentially expressed genes (CDEGs) between the two datasets were illustrated using a Venn diagram. To identify key genes, univariate Cox regression analysis was performed to assess the association between CDEG expression and overall survival (OS) in AML samples from the TCGA-LAML dataset. Genes with P values < 0.05 in the TCGA-LAML dataset underwent correlation analysis. Based on the correlation analysis results, NAP1L5 was selected as the target gene for subsequent analysis. Subsequently, box scatter plots were generated to further evaluate NAP1L5 expression across the three datasets. Additionally, within the TCGA dataset, samples were stratified based on the median expression level of NAP1L5, and Kaplan-Meier (K-M) survival curves were used to assess the association between different NAP1L5 expression levels and AML prognosis. All visualizations, including volcano plots, Venn diagrams, and box scatter plots, were created using the ggplot2 package. K-M curve analysis and univariate Cox regression analysis were conducted using the survival and survminer packages, respectively.

### 2.3 Analysis of differentially expressed genes for NAP1L5

AML patients in the TCGA-LAML dataset were stratified into high- and low-expression groups based on median NAP1L5 expression levels. Using the DESeq2 package (18) with thresholds of |logFC| > 1 and P.value < 0.05, DEGs between NAP1L5 high- and low-expression groups were identified and visualized via volcano plots, where genes meeting logFC > 1 and P.value < 0.05 were defined as upregulated DEGs, while those with logFC < -1 and P.value < 0.05 were downregulated DEGs. Additionally, Spearman correlation coefficients between CDEGs and NAP1L5 expression levels were calculated, and the top 20 genes exhibiting the strongest absolute correlation with the target gene NAP1L5 were selected in descending order of |logFC| for subsequent co-expression analysis, with results presented in a heatmap generated using the ggplot2 package.

### 2.4 Enrichment analysis

Gene Set Enrichment Analysis (GSEA) was performed to identify potential biological pathways associated with high and low NAP1L5 expression groups. The analysis was conducted with a seed value of 2022, 10,000 permutations, and gene set size constraints of 10 to 500 genes. The reference gene set used was c2.cp.all.v2022.1.Hs.symbols.gmt. To elucidate the biological processes involving NAP1L5, Gene Ontology (GO) enrichment analysis was performed on NAP1L5 and the top 20 genes with the highest correlation. Significant enrichment was defined by an adjusted p-value (P.adj) < 0.05 and a false discovery rate (FDR, q-value) < 0.05. Both GSEA and GO analyses were carried out using the clusterProfiler package (19).

### 2.5 Interaction analysis of NAP1L5

Applying the STRING database (http://string-db.org/) (20), the protein-protein interaction (PPI) network of NAP1L5 was constructed with a minimum interaction score of medium confidence (0.400). The miRDB database (21) was utilized to predict the mRNA-miRNA interaction network of NAP1L5 (target scores greater than 80 were utilized for visualization). Using the ChIPBase database (22) (version 3.0) (https://rna.sysu.edu.cn/chipbase/), transcription factors (TFs) binding to NAP1L5 were identified (visualization was limited to results with a sample size > 2 in the ChIPBase database). RNA-binding proteins (RBPs) that interact with NAP1L5 were predicted using the ENCORI database (23) (https://starbase.sysu.edu.cn/). The results were visualized with Cytoscape version 3.9.1 (24).

### 2.6 Investigating the correlation between NAP1L5 expression and immune infiltration in AML

To explore the relationship between NAP1L5 expression and immune infiltration in AML, the LM22 signature gene matrix and matrix data from the high-expression and low-expression NAP1L5 groups in the TCGA-LAML dataset were integrated using the CIBERSORT platform (25). Data were filtered to include only samples with an immune cell enrichment score > 0. Spearman’s correlation was employed to assess the relationship between immune cell infiltration levels across different subgroups. Additionally, the Tumor Immune Dysfunction and Exclusion (TIDE) score and Tumor Mutation Burden (TMB) were used to evaluate the potential correlation between NAP1L5 expression levels and immune therapy response.

### 2.7 Nomogram construction and validation

To further determine the prognostic value of NAP1L5 in AML, we first performed univariate Cox regression analysis on NAP1L5 expression levels and relevant clinical information (FAB, gender, age) in AML patients from the TCGA-LAML dataset, subsequently incorporating variables with P-value < 0.1 from the univariate analysis into multivariate Cox regression to identify independent prognostic factors for nomogram construction. We then extracted coefficients (lambda) of each variable in the clinical prognostic model to calculate the Risk Score, ranked and stratified patients by median Risk Score, and generated risk factor plots along with prognostic KM curves for high/low Risk Score groups, with the formula as follows:


$$\text{Risk Score }=\;\sum_{\text{i}}\text{Coefficient }\left({\text{hub gene}}_{\text{i}}\right)*\text{mRNA Expression }\left({\text{hub gene}}_{\text{i}}\right)$$

Finally, the nomogram’s accuracy and discriminative power were evaluated via calibration curves (26) and decision curve analysis (DCA), while time-dependent ROC curves (27) assessed the diagnostic efficacy of Risk Score stratification for AML patient survival. Visualizations were created using R packages “rms” (28), “ggDCA” (29), “timeROC” (30), “survival”, and “survminer”.

### 2.8 Investigating the correlation between NAP1L5 expression and drug sensitivity

The correlation between NAP1L5 expression levels and drug sensitivity was analyzed using drug information from the Genomics of Drug Sensitivity in Cancer (GDSC) database (31) (https://www.cancerRxgene.org).

### 2.9 Sample collection

Peripheral blood samples were collected from 100 AML patients treated at the Department of Hematology, The First Affiliated Hospital of Bengbu Medical University, and 20 healthy donors between 2022 and 2023. All samples were analyzed in compliance with the principles of the Declaration of Helsinki, under the supervision of the hospital’s ethics review committee, and after obtaining written informed consent from all participants.

### 2.10 Cell sorting, culture, and transfection

A CD34+ cell isolation kit (MACS) was used in this study to separate CD34+ cells from healthy volunteers bone marrow. Hasenbio (Wuxi, China) provided the human myeloid leukemia cell lines HL60, U937, Kasumi-6, and KG-1. 10% fetal bovine serum (FBS; Thermo Fisher Scientific, Inc.) was added to RPMI-1640 medium (Gibco, USA) for the culture of all cell lines. For the following experiments, the HL60 cell line was chosen. GenePharma created the sh-NAP1L5 plasmid, overexpression (OE) NAP1L5 plasmids, and negative control constructs.

The following were the sequences of sh-NAP1L5: 5’-GCGATAAGATAGAAGCCAAAT-3’; 5’-GAAGCGATGCGATAAGATAGA-3’ and 5’-CAGAAGCGATGCGATAAGATA-3’ corresponding to Sh-NAP1L5-1, Sh-NAP1L5-2, and Sh-NAP1L5-3, respectively. The efficiency of transfection was assessed in subsequent experiments after cells were transfected with shRNAs or overexpression plasmids using Lipofectamine 2000 (Invitrogen, USA) in accordance with the manufacturer’s instructions.

### 2.11 Quantitative RT-qPCR analysis

Biosharp Trizol reagent was used to extract total RNA, and qPCR SYBR Green Master Mix (No Rox) (Cronda, Shanghai, China) and a one-step cDNA synthesis kit were used to measure the levels of mRNA expression. β-actin was used as an internal reference gene. Relative gene expression levels were measured using the 2^-ΔΔCt^ method. **Supplementary Table S2** provides the primer sequences.

### 2.12 Western blot assay

Total protein was extracted using RIPA lysis buffer (Biosharp, Hefei, China). Protein concentration was determined using the BCA method (Biosharp, Hefei, China). A total of 30 μg of protein was separated by SDS-PAGE and subsequently transferred to a PVDF membrane. After blocking the membrane with 5% BSA at room temperature, primary antibodies against NAP1L5 (1:1000, Invitrogen) and β-actin (1:2000, Servicebio) were added and incubated overnight at 4°C. The protein expression on the membrane was visualized using ECL following incubation with secondary antibodies, namely anti-rabbit HRP (1:4000, Biosharp).

### 2.13 Cell proliferation and apoptosis assays

Six-well and 96-well plates were seeded with HL60 cells at densities of 1×10^5^ and 3×10^4^ cells/well, respectively. After 48 hours of culture following transfection, the rate of cell proliferation and absorbance were assessed using CCK-8 (Biosharp, Hefei, China) and 450 nm. The Annexin V-FITC/PI apoptosis detection kit (Cronda, Shanghai, China) was used for flow cytometry (FCM) to confirm the apoptosis rate.

### 2.14 Transwell assay

HL60 cells were seeded at a density of 5×10^4^ cells/mL in 24-well plates (200 μL per well) in the upper chamber, with 800 μL of medium containing 20% FBS (Thermo Fisher, Inc.) added to the lower chamber. After incubation for 48 hours, non-migrated cells were removed. Subsequently, migrated cells on the bottom of the lower chamber were fixed, stained, and imaged.

### 2.15 Colony formation assay

Plates with six wells were seeded with 4x10^5^ transfected HL60 cells. Crystal violet staining was applied to the colonies after two to three weeks. An inverted microscope was used to take pictures after the experiment.

### 2.16 Statistical analysis

Data processing and analysis were conducted using GraphPad Prism 9.5.1 and R software (version 4.2.2). Comparisons between two groups were performed using the independent Student’s t-test and the Wilcoxon rank-sum test. Survival analysis was carried out using Cox regression and Kaplan-Meier survival curves. Spearman correlation analysis was employed to determine correlation coefficients. Data, derived from at least three independent experiments, are presented as mean ± standard deviation (SD). Statistical significance was set at P < 0.05, with the following thresholds: *, P < 0.05; **, P < 0.01; ***, P < 0.001; ****, P < 0.0001; N.S., not significant.

## 3 Results

### 3.1 Identification of differentially expressed genes and association of NAP1L5 with AML

**Figure 1:**
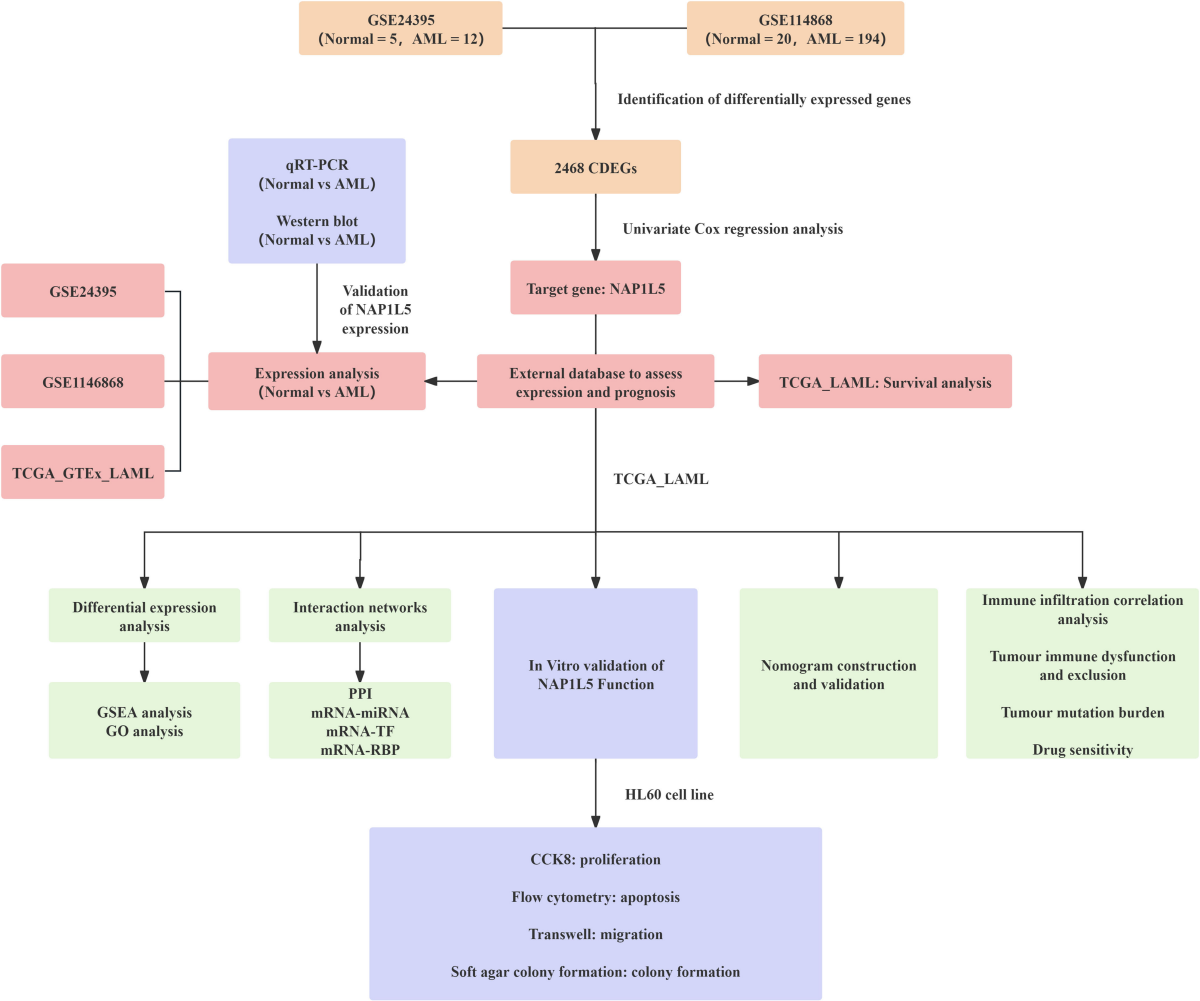
This study’s research flow chart.

**Figure 2:**
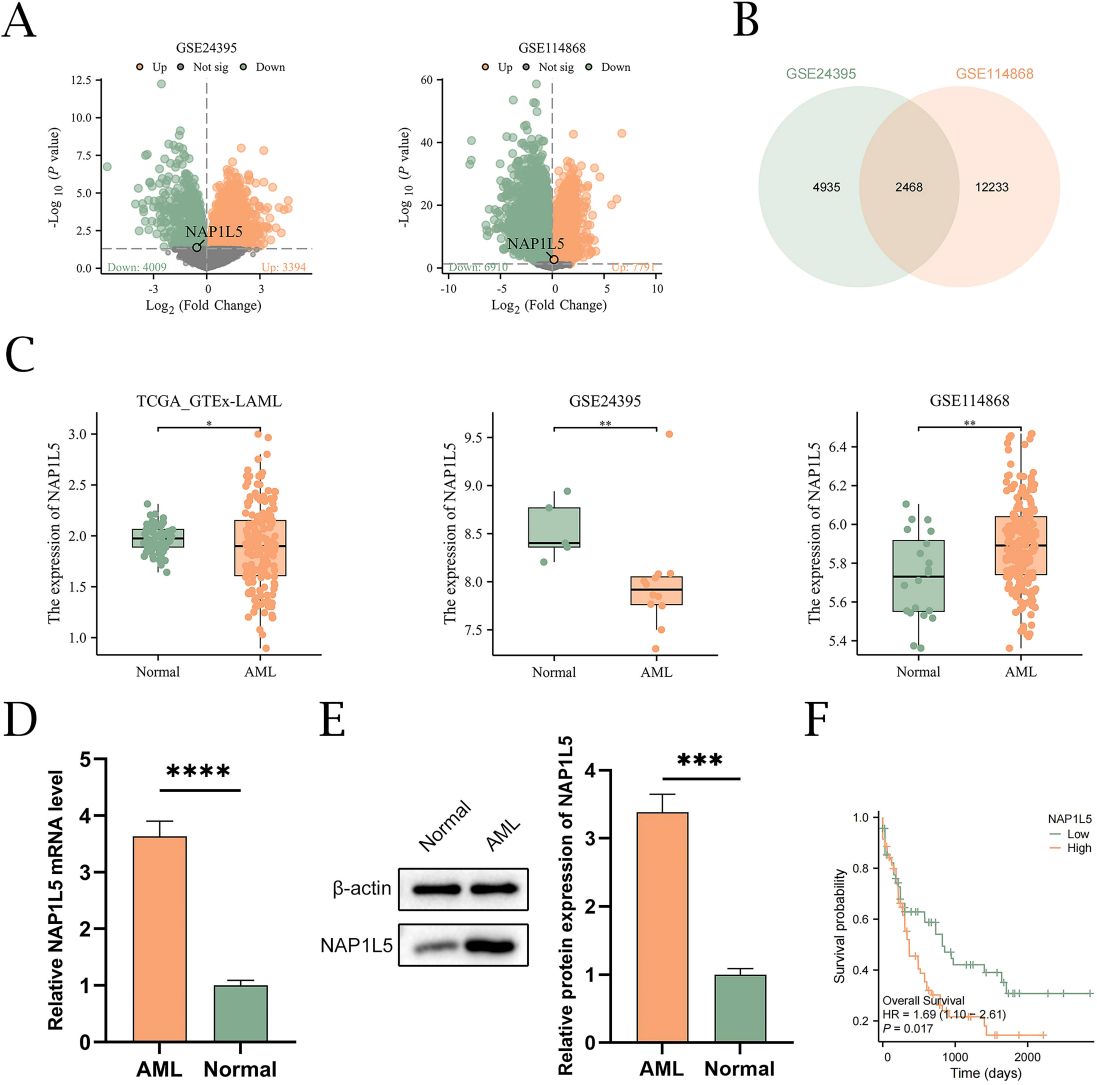
Identification of differentially expressed genes and association of NAP1L5 with AML. **(A)** Volcano plots showing DEGs from the GSE24395 and GSE114868 datasets. **(B)** Venn diagram showing the CDEGs from the GSE24395 and GSE114868 datasets. **(C)** Differential expression analysis of NAP1L5 in AML patients and healthy controls from the TCGA_GTEx-LAML, GSE24395, and GSE114868 datasets. **(D, E)** Expression analysis of NAP1L5 mRNA and protein in AML patients using qRT-PCR and Western blot, respectively. **(F)** K-M curves for OS of AML patients with different NAP1L5 expression levels in the TCGA-LAML database. ***, P < 0.001; ****, P < 0.0001.

The integrated research workflow is depicted in **Figure 1**. Differential expression analysis of the GSE24395 and GSE114868 datasets was performed using the limma package (**Figure 2A**). In GSE24395, we identified 7,403 differentially expressed genes (DEGs) (P<0.05), comprising 3,394 upregulated (logFC > 0) and 4,009 downregulated (logFC < 0) genes. Similarly, GSE114868 yielded 14,701 DEGs, with 7,791 upregulated and 6,910 downregulated genes. Intersection analysis of DEGs from both datasets (|logFC| > 0 and P < 0.05) revealed 2,468 common DEGs (CDEGs) (**Figure 2B**; **Supplementary Table S3**). To investigate the association between these CDEGs and prognosis in acute myeloid leukemia (AML), univariate Cox regression analysis was conducted using the TCGA-LAML dataset. Results (**Supplementary Table S4**) indicated significant correlations between the expression levels of several CDEGs and overall survival (OS), identifying NAP1L5 as a promising candidate for further investigation. Subsequent analysis of NAP1L5 expression levels across the TCGA-LAML, GSE24395, and GSE114868 datasets showed significant differences between AML patients and healthy controls in all three (**Figure 2C**), although the observed trends appeared inconsistent. To validate NAP1L5 expression in AML, peripheral blood samples were collected from patients and healthy individuals. Analysis by Western blotting and qRT-PCR confirmed significantly elevated NAP1L5 mRNA and protein levels in the AML group compared to the healthy group (**Figures 2D, E**). Furthermore, Kaplan-Meier (K-M) curve analysis demonstrated that AML patients with high NAP1L5 expression in the TCGA-LAML dataset had a poorer prognosis (**Figure 2F**).

### 3.2 GSEA reveals pathways associated with NAP1L5 in AML

**Figure 3:**
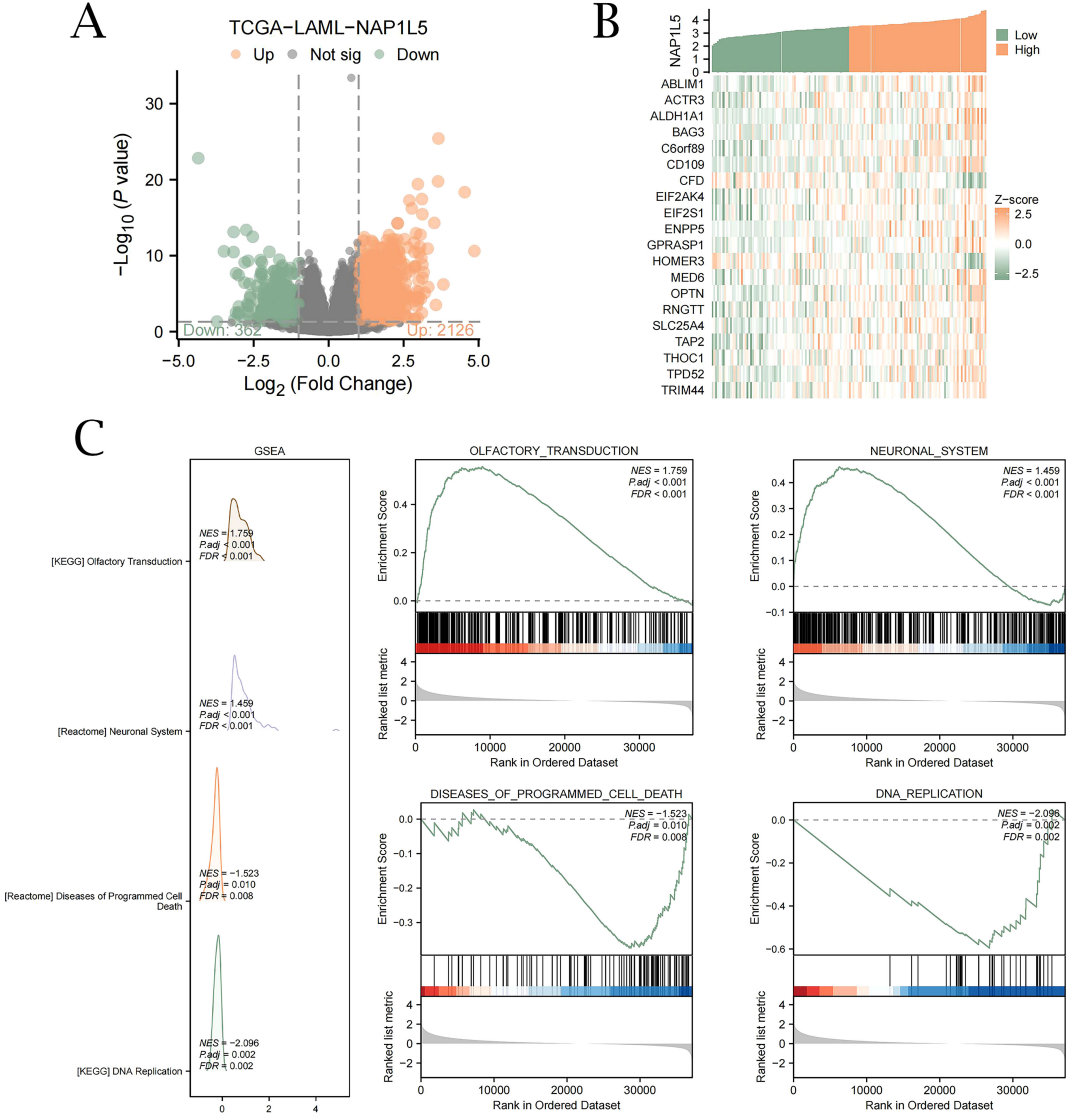
Differential expression gene analysis and GSEA of NAP1L5 in TCGA. **(A)** Analysis of single-gene differences between groups in the TCGA-LAML dataset with high and low NAP1L5 expression. **(B)** Heatmap of 20 genes co-expressed with NAP1L5. **(C)** GSEA enrichment analysis reveals four key biological functions primarily involved in NAP1L5 and its enrichment in olfactory transduction, nervous system, apoptosis, and DNA replication pathways. (FDR, false discovery rate; NES, normalized enrichment score; Significant enrichment screening criteria were set at P < 0.05 and FDR P < 0.05).

We first performed differential expression analysis on TCGA-LAML and identified 2,126 upregulated genes and 362 downregulated genes that were co-expressed with NAP1L5 (**Figure 3A**). Subsequently, Spearman correlation coefficients between CDEGs and NAP1L5 expression levels were calculated, and the top 20 genes exhibiting the strongest absolute correlation with the target gene NAP1L5 were selected in descending order of |logFC|. Based on the top 18 upregulated DEGs and the top 2 downregulated DEGs, we constructed a co-expression heatmap (**Figure 3B**). Furthermore, GSEA analysis results further indicated that NAP1L5 expression may regulate the development of AML by affecting olfactory signaling pathways, nervous system signaling pathways, apoptotic pathways, and DNA replication pathways (**Figure 3C**, listed in **Supplementary Table S5**).

### 3.3 Functional enrichment analysis

**Figure 4:**
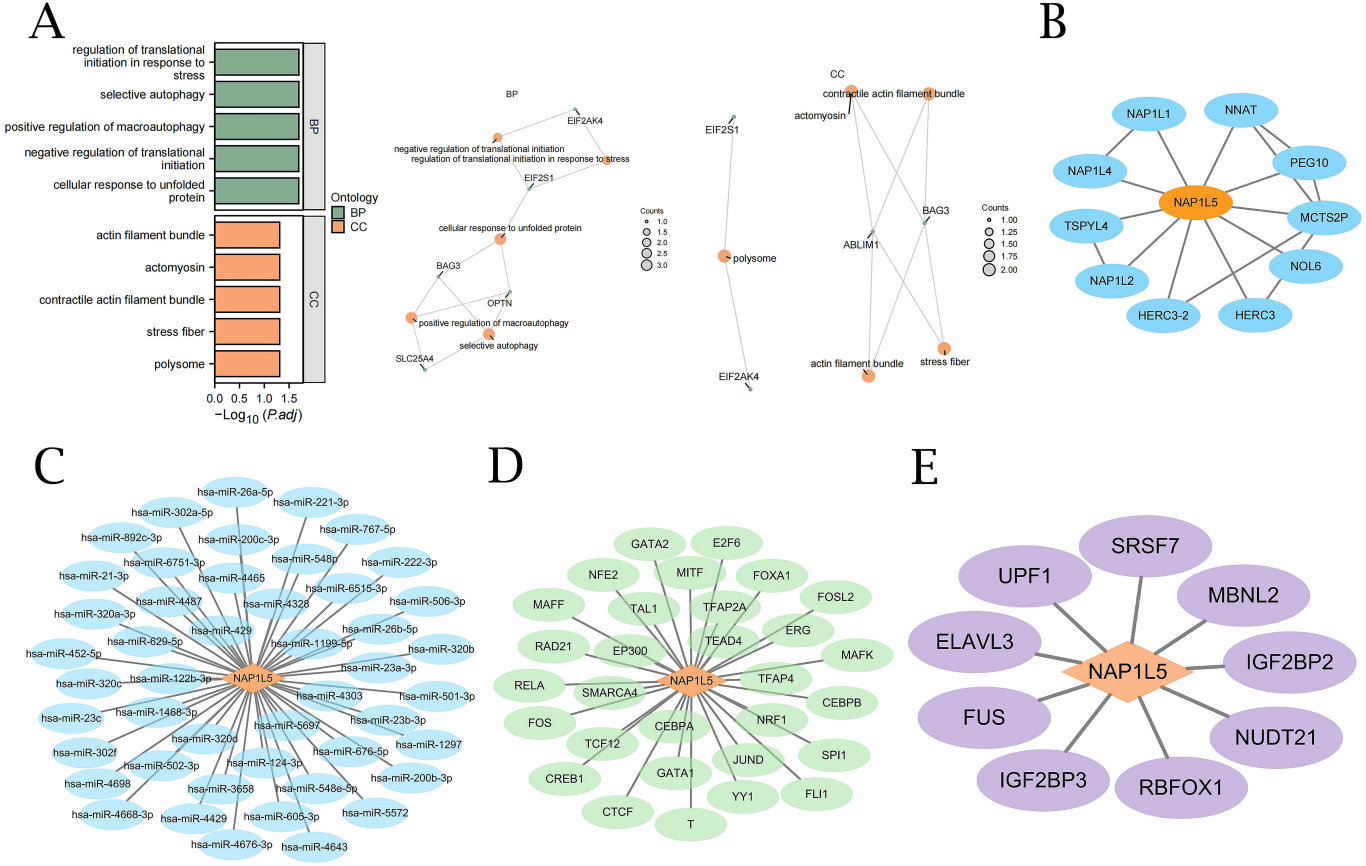
GO enrichment and interaction network analysis of NAP1L5. **(A)** GO analysis of NAP1L5 and 20 related genes. **(B)** NAP1L5’s Protein-Protein Interaction (PPI) network. **(C)** NAP1L5’s mRNA-miRNA network. **(D)** NAP1L5’s mRNA-TF network. **(E)** NAP1L5’s mRNA-RBP network.

To elucidate the biological role of NAP1L5 in AML, we conducted Gene Ontology (GO) enrichment analysis on 20 genes identified as being associated with NAP1L5 (**Supplementary Table S6**). The analysis revealed significant enrichment in biological processes (BP) related to cellular stress response and protein synthesis regulation, including autophagy, translational initiation regulation in response to stress, cellular response to unfolded protein, and negative regulation of translational initiation. Furthermore, at the cellular component (CC) level, these genes were predominantly associated with structures involved in cellular structure and function, such as macromolecular complexes, stress fibers, contractile actin filaments, myosin, and actin filaments (**Figure 4A**).

### 3.4 Interaction network analysis

Using a variety of databases, we conducted in-depth analyses of the protein-protein interaction (PPI), mRNA-miRNA, mRNA-TF, and mRNA-RBP interaction networks in order to fully understand the molecular regulatory network of NAP1L5. STRING database analysis revealed interactions between NAP1L5 and 10 genes, including NAP1L1, NNAT, PEG10, MCTS2P, NOL6, HERC3, HERC3-2, NAP1L2, NAP1L4, and TSPYL4 (**Figure 4B**). miRDB database analysis identified 47 miRNA molecules associated with NAP1L5 (**Figure 4C**, listed in **Supplementary Table 7**). ChIPBase database analysis revealed 30 transcription factors (TFs) associated with NAP1L5 (**Figure 4D**, listed in **Supplementary Table 8**). ENCORI database analysis showed that NAP1L5 is associated with 9 RNA-binding protein (RBP) molecules (**Figure 4E**, listed in **Supplementary Table 9**).

### 3.5 Immune infiltration analysis of NAP1L5

**Figure 5:**
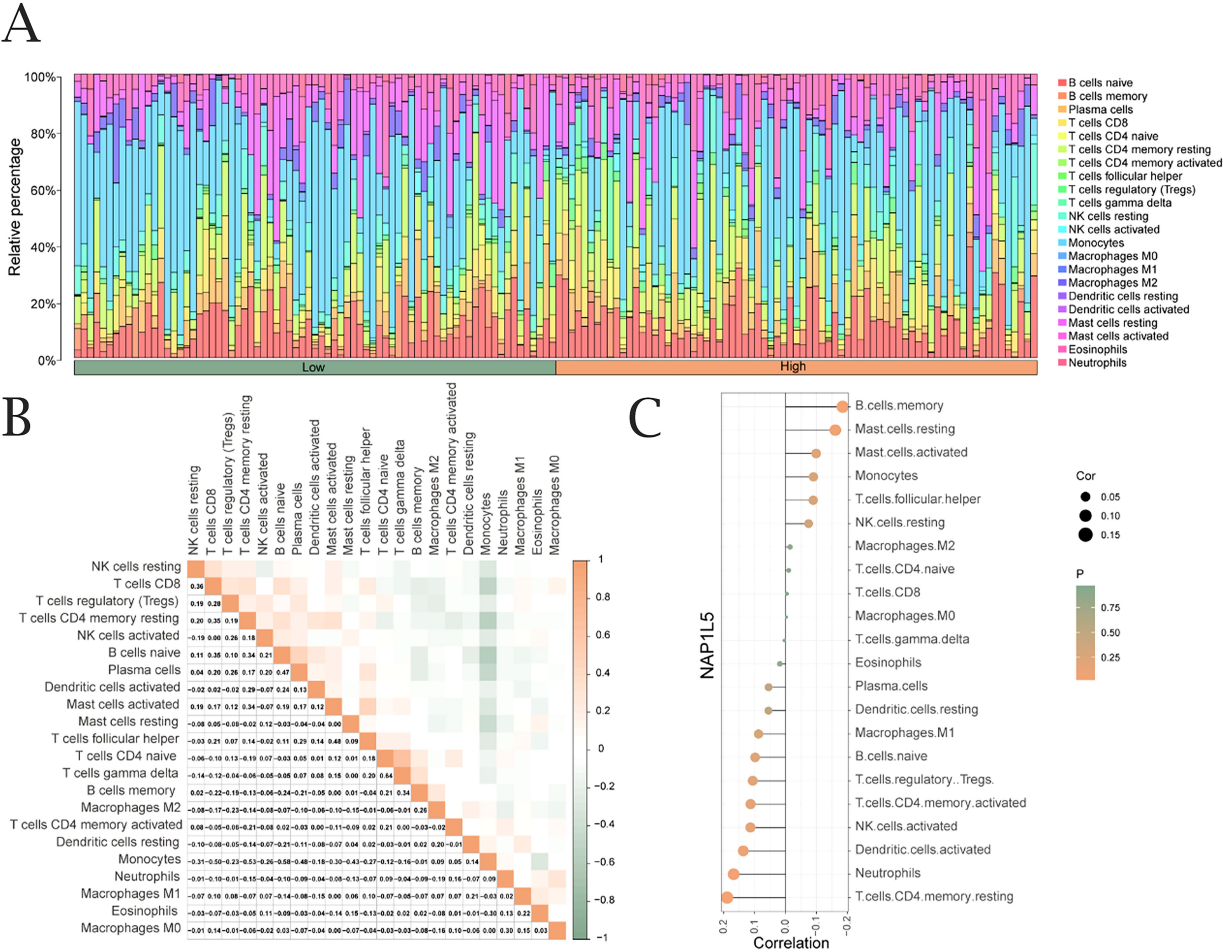
Immune infiltration analysis of NAP1L5. **(A)** Correlation between NAP1L5 expression levels and immune infiltration (CIBERSORT algorithm). **(B)** Correlation analysis of immune cell infiltration abundance. **(C)** Lollipop plot of immune cell correlations with NAP1L5 in TCGA-LAML dataset.

Using the CIBERSORT algorithm, we analyzed the correlation between NAP1L5 expression levels in AML (TCGA-LAML dataset) and the infiltration abundance of 22 immune cell types. The results revealed significant correlations, with both positive and negative associations observed (**Figure 5A**). Notably, the overall correlation trend leaned slightly stronger toward negative associations between NAP1L5 and the 22 immune cells (**Figure 5B**). Among the immune cell types, T cells CD4 memory resting showed the highest positive correlation with NAP1L5, while B cell memory exhibited the strongest negative correlation (**Figure 5C**).

### 3.6 Nomogram construction and validation

**Figure 6:**
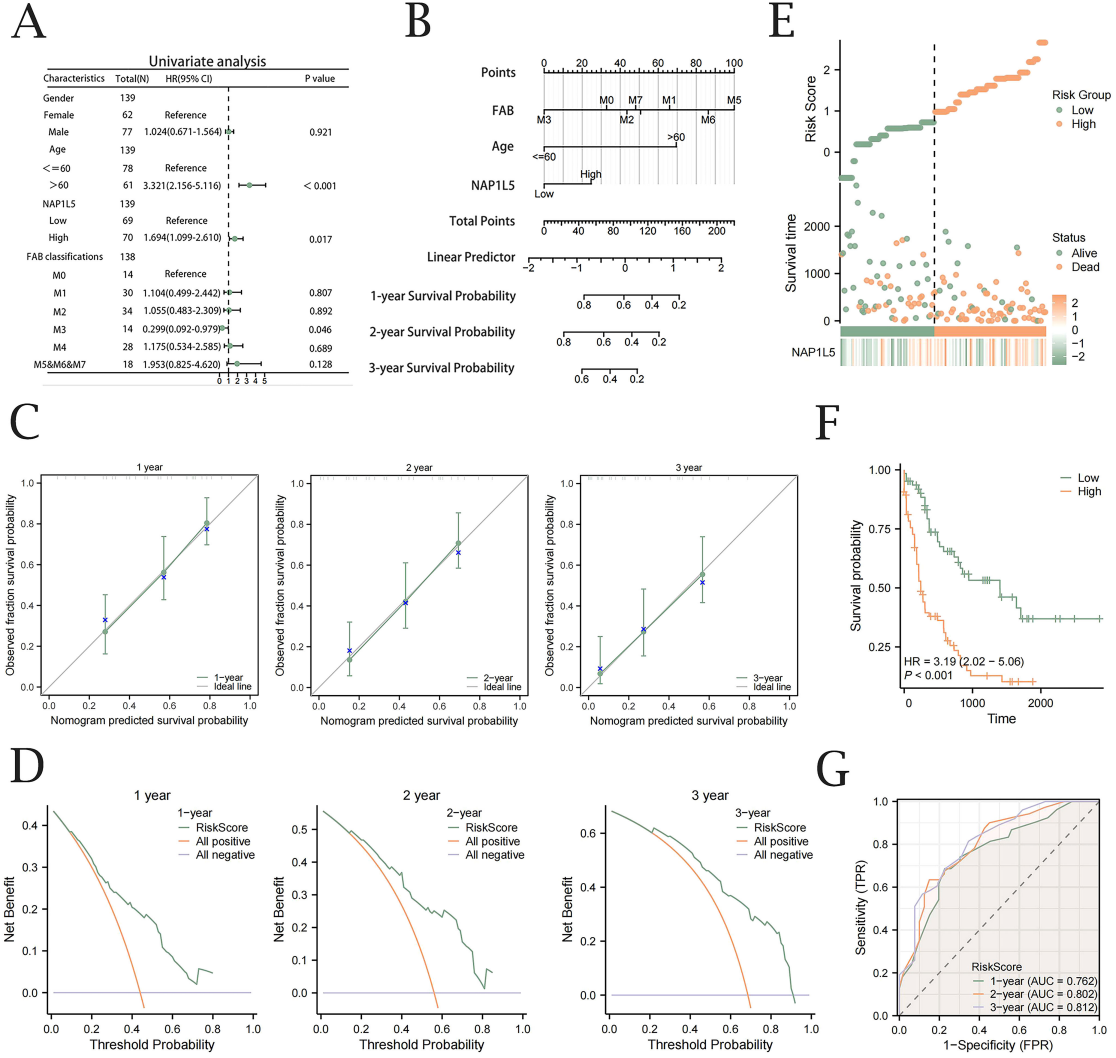
Nomogram construction and validation. **(A)** Forest plot of univariable Cox regression analysis integrating NAP1L5 expression levels and associated clinical factors. **(B)** Nomograph prediction model based on NAP1L5 expression levels and associated clinical features. **(C)** Calibration curves with one, two, and three years for the Cox regression prediction model. **(D)** DCA plots for the Cox regression prognostic model at one, two, and three years. **(E)** Clinical prognostic model risk score plot. **(F)** Prognostic KM survival curves stratified by median Risk Score. **(G)** Time-dependent ROC analysis evaluating the diagnostic performance of Risk Score stratification for AML patient survival. (All based on the TCGA-LAML database).

Univariate Cox regression analysis revealed that AML patient prognosis was significantly correlated with NAP1L5 expression levels, age, and FAB classification (**Figure 6A**, **Supplementary Table 10**). To construct a prognostic model for AML, we integrated NAP1L5 expression levels with relevant clinical characteristics. The model demonstrated that FAB classification had the highest predictive value (**Figure 6B**). Multivariable analysis further confirmed the model’s predictive performance. Prognostic calibration curve analysis indicated that the model accurately predicted 1-year, 2-year, and 3-year survival rates for AML patients, with prediction accuracy improving over time (**Figure 6C**). Decision curve analysis (DCA) further validated the model’s clinical utility, showing increasing application value at 1-year, 2-year, and 3-year intervals (**Figure 6D**). Risk factor plots visually summarized the model’s prediction results (**Figure 6E**). The Kaplan-Meier survival curve analysis further supported the model’s findings, revealing a significant difference in prognosis between AML patients grouped by median risk score (P < 0.001, **Figure 6F**). Time-dependent ROC curve analysis further confirmed the strong diagnostic performance of the clinical prognostic model’s risk score in predicting AML patient outcomes (**Figure 6G**).

### 3.7 Immunological evaluation and drug sensitivity analysis of NAP1L5

**Figure 7:**
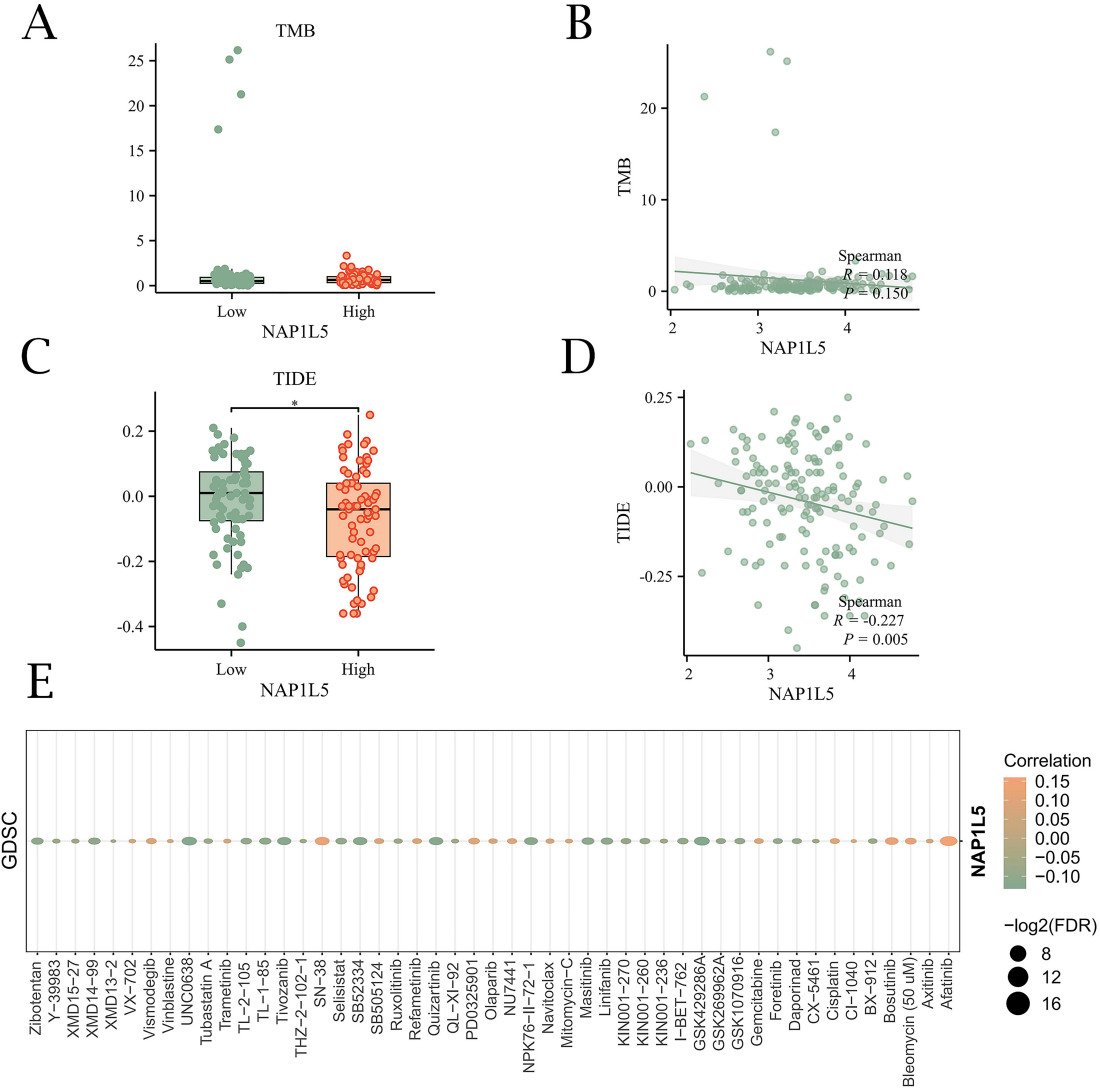
Immunological evaluation and drug sensitivity analysis of NAP1L5. **(A, B)** Relationship between NAP1L5 expression levels and TMB. **(C, D)** Relationship between NAP1L5 expression levels and TIDE score. **(E)** Relationship between NAP1L5 expression levels and drug sensitivity.

To explore the potential role of NAP1L5 in AML immunotherapy, we investigated its association with tumor immune dysfunction and exclusion (TIDE) scores and tumor mutational burden (TMB). Correlation analysis revealed that TMB did not show statistically significant differences between subgroups (P > 0.05) (**Figures 7A, B**). However, TIDE scores were significantly different between high and low NAP1L5 expression subgroups in the TCGA-LAML dataset (P < 0.05) (**Figures 7C, D**). Additionally, drug sensitivity analysis using the GDSC database identified 49 drugs interacting with NAP1L5. Most of these interactions exhibited negative correlations with NAP1L5 expression (**Figure 7E**).

### 3.8 Functional validation of NAP1L5 in AML through *in vitro* experiments

**Figure 8:**
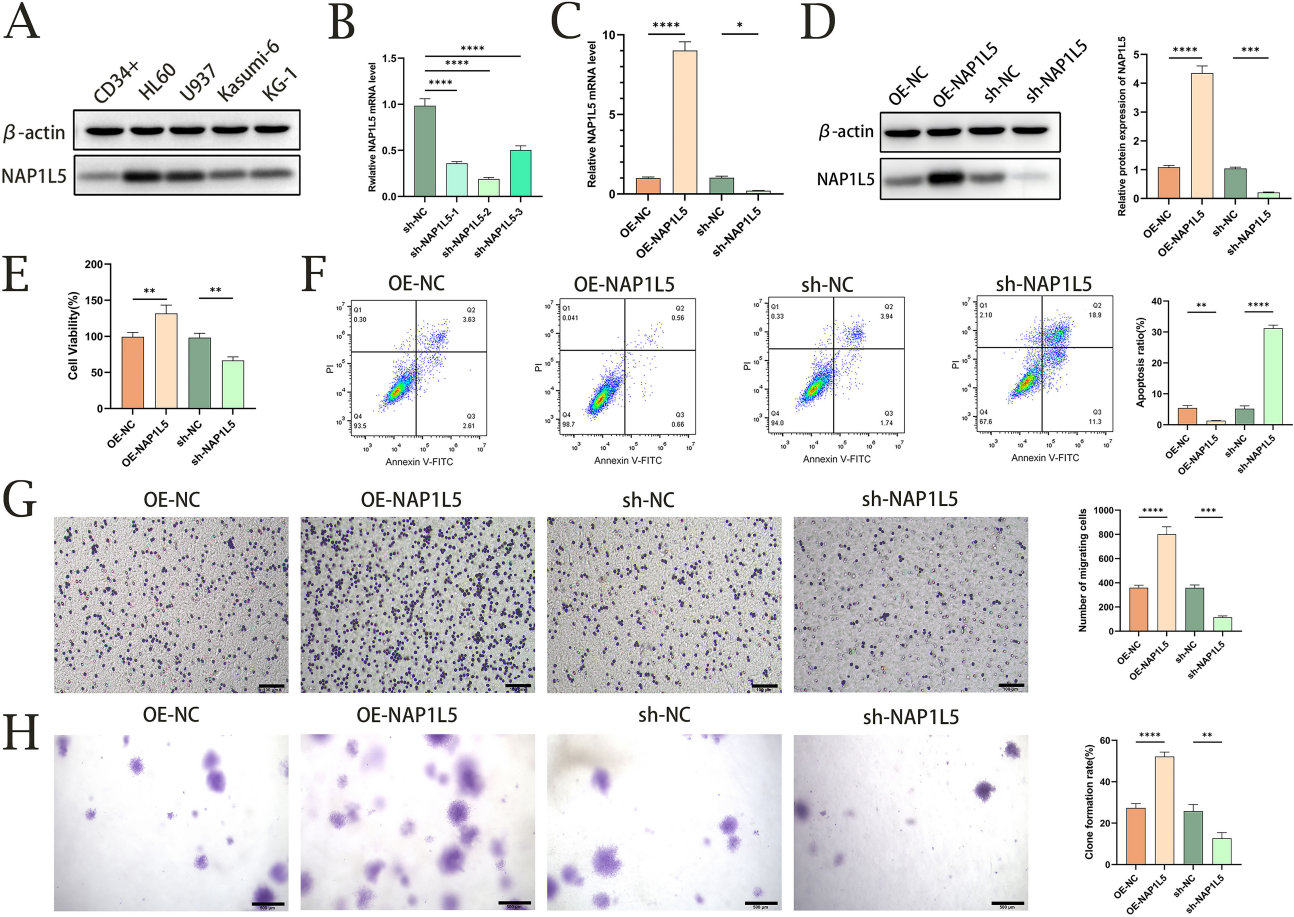
Functional validation of NAP1L5 in AML through *in vitro* experiments. **(A)** Western blot analysis of NAP1L5 protein expression in HL60, U937, Kasumi-6, and KG-1 cell lines. **(B)** Validation of NAP1L5 knockdown efficiency in HL60 cells using lentiviral vectors expressing shRNA targeting NAP1L5 (sh-NAP1L5-1, sh-NAP1L5-2, sh-NAP1L5-3). **(C, D)** Confirmation of NAP1L5 mRNA and protein expression levels in HL60 cells following knockdown or overexpression. **(E)** CCK8 assay assessing the impact of NAP1L5 overexpression or silencing on HL60 cell proliferation. **(F)** Flow cytometry analysis evaluating the effect of NAP1L5 on HL60 cell apoptosis. **(G)** Transwell assay examining the influence of NAP1L5 on HL60 cell migration. **(H)** Soft agar colony formation assay assessing the effect of NAP1L5 on HL60 cell colony formation. *, P < 0.05; **, P < 0.01; ***, P < 0.001; ****, P < 0.0001.

We investigated several AML cell lines in order to confirm the connection between NAP1L5 and AML. We discovered that all AML cell lines had significantly higher NAP1L5 protein expression levels when compared to CD34+ cells as a control, with HL60 showing the largest increase (**Figure 8A**). HL60 was thus selected for further research. Subsequently, we generated three HL60 cell lines with NAP1L5 knockdown. Among these, the sh-NAP1L5–2 cell line exhibited the lowest mRNA expression level of NAP1L5 (**Figure 8B**) and was chosen for subsequent studies. The stability of NAP1L5 knockdown and overexpression was confirmed by qRT-PCR and Western blot (**Figures 8C, D**). Overexpression of NAP1L5 markedly promoted AML cell proliferation, while attenuation of NAP1L5 expression significantly inhibited cell proliferation (**Figure 8E**). Regarding apoptosis, the Annexin-V-APC/PI assay demonstrated that NAP1L5 overexpression notably inhibited apoptosis, whereas silencing NAP1L5 resulted in the opposite effect (**Figure 8F**). Additionally, NAP1L5 overexpression promoted cell migration and colony formation, while silencing NAP1L5 remarkably inhibited these processes (**Figures 8G, H**).

## 4 Discussion

In recent years, advancements in AML treatment modalities—including traditional chemotherapy, hypomethylating agents, targeted drugs, and stem cell transplantation—have gradually improved patient survival rates (32). However, significant challenges remain, such as resistance to initial treatments and disease recurrence (33). Enhancing the prognosis of AML patients remains a critical priority.

NAP1L5, a protein that forms complexes with histones, plays a crucial role in histone transport, preventing histone accumulation and ensuring orderly nucleosome formation (34). Notably, NAP1L5 is paternally imprinted in mouse single embryos (35). As an oncogene, NAP1L5 is involved in cell division, cellular aging, and tumorigenesis, promoting tumor cell survival, proliferation, colony formation, and invasion. Its aberrant expression or mutation has been linked to the development and progression of certain cancers (36). Thus, exploring NAP1L5 may provide insights into nucleosome assembly and DNA repair mechanisms, potentially offering novel therapeutic avenues for cancer treatment.

In this study, NAP1L5 was identified as a target gene based on Common Differentially Expressed Genes (CDEGs). GSEA and Gene Ontology (GO) enrichment analyses were performed on NAP1L5 and its 20 most correlated genes. A prognostic model was constructed using COX regression, and drug sensitivity analysis was conducted for NAP1L5. Immune cell type scores, transcription factor exploration, and drug sensitivity analysis were also performed. GSEA revealed that NAP1L5 expression predominantly impacts olfactory signal transduction, neural signal transduction, programmed cell death, and DNA replication pathways in AML, suggesting its involvement in multiple signaling pathways that regulate disease onset and progression. Interactions between NAP1L5 and multiple microRNAs (miRNAs), transcription factors (TFs), and RNA-binding proteins (RBPs) were identified, highlighting its diverse biological roles in AML. Immune infiltration analysis showed a positive correlation between NAP1L5 and T cell CD4 memory resting, while B cell memory exhibited a negative correlation, potentially influencing immunoregulatory functions in AML patients. Drug sensitivity analysis revealed associations between NAP1L5 and multiple drug resistances, indicating poor prognosis for AML patients with high NAP1L5 expression. Enrichment analysis identified 20 genes associated with NAP1L5, including BAG3 and HOMER3. BAG3, a member of the BAG family, regulates tumor cell adhesion, migration, and invasion, promoting recurrence and metastasis (37). It is positively correlated with activated memory CD4+ T cells and poorer overall survival (OS) in AML patients (38). Our findings align with these studies, showing a significant correlation between BAG3 and NAP1L5, as well as a high positive correlation between NAP1L5 and CD4 resting memory T cells, which are linked to shorter OS in AML patients. Similarly, HOMER3, markedly overexpressed in childhood AML, serves as a potential biomarker and therapeutic target (39). Its significant correlation with NAP1L5 further supports the findings of this study. Our experimental results demonstrated that NAP1L5 is upregulated in AML patients at both mRNA and protein levels, a finding confirmed in multiple AML cell lines. Functional assays revealed that NAP1L5 overexpression promotes cell proliferation, inhibits apoptosis, enhances cell migration, and facilitates colony formation, while its knockdown has the opposite effect.

In conclusion, this study highlights the significant role of NAP1L5 in promoting AML development. However, limitations include the use of a single cell line and the lack of detailed mechanistic exploration into how NAP1L5 influences AML progression. Future work should focus on additional experiments to further validate the role of NAP1L5 in AML and elucidate its underlying mechanisms.

## Data Availability

The original contributions presented in the study are included in the article/**Supplementary Material**. Further inquiries can be directed to the corresponding authors.
